# A novel antagonist to the inhibitors of apoptosis (IAPs) potentiates cell death in EGFR-overexpressing non-small-cell lung cancer cells

**DOI:** 10.1038/cddis.2014.447

**Published:** 2014-10-16

**Authors:** S-H Lee, J-Y Lee, C L Jung, I H Bae, K H Suh, Y G Ahn, D-H Jin, T W Kim, Y-A Suh, S J Jang

**Affiliations:** 1 Institute for Innovative Cancer Research, Asan Institute for Life Science, Seoul Asan Medical Center, The University of Ulsan College of Medicine, Seoul, Republic of Korea; 2 Hanmi Research Center, Hanmi Pharm. Co., Ltd., Hwaseong, Gyeonggi-do, Republic of Korea; 3 Department of Medicinal Oncology, Seoul Asan Medical Center, The University of Ulsan College of Medicine, Seoul, Republic of Korea; 4 Department of Pathology, Seoul Asan Medical Center, The University of Ulsan College of Medicine, Seoul, Republic of Korea

## Abstract

In the effort to develop an efficient chemotherapy drug for the treatment of non-small-cell lung cancer (NSCLC), we analyzed the anti-tumorigenic effects of a novel small molecule targeting the inhibitor of apoptosis (IAPs), HM90822B, on NSCLC cells. HM90822B efficiently decreased IAP expression, especially that of XIAP and survivin, in several NSCLC cells. Interestingly, cells overexpressing epidermal growth factor receptor (EGFR) due to the mutations were more sensitive to HM90822B, undergoing cell cycle arrest and apoptosis when treated. In xenograft experiments, inoculated EGFR-overexpressing NSCLC cells showed tumor regression when treated with the inhibitor, demonstrating the chemotherapeutic potential of this agent. Mechanistically, decreased levels of EGFR, Akt and phospho-MAPKs were observed in inhibitor-treated PC-9 cells on phosphorylation array and western blotting analysis, indicating that the reagent inhibited cell growth by preventing critical cell survival signaling pathways. In addition, gene-specific knockdown studies against XIAP and/or EGFR further uncovered the involvement of Akt and MAPK pathways in HM90822B-mediated downregulation of NSCLC cell growth. Together, these results support that HM90822B is a promising candidate to be developed as lung tumor chemotherapeutics by targeting oncogenic activities of IAP together with inhibiting cell survival signaling pathways.

Resistance to apoptosis is a hallmark of many solid tumors, including lung cancer, and is, therefore, an important target mechanism for controlling cancer proliferation. The inhibitor of apoptosis (IAP) is a family of proteins containing one or more conserved cysteine and histidine-rich baculoviral IAP repeat (BIR) in their N-terminal domains and a C-terminal RING (really interesting new gene) domain. The BIR domains of IAPs form zinc figure-like structures that bind to active caspases to block caspase activity, while the RING domain acts as an ubiquitin ligase to facilitate proteasome degradation of caspases. Several IAPs have been identified in mammals, including X-linked IAP (XIAP), cellular IAP-1 and -2 (cIAP-1 and cIAP-2) and survivin. Among these IAP proteins, XIAP is a central regulator of both the death receptor- and mitochondria-mediated apoptosis pathways. Consistent with their role in the inhibition of apoptosis, XIAP and survivin are highly expressed in a diverse array of tumors and are often associated with resistance to apoptosis and low sensitivity to chemotherapy drugs in some tumor types.^1, 2, 3^

Recent studies have shown that inhibition of the expression level or function of survivin and/or XIAP with anti-sense RNA, short interfering RNA (siRNA), dominant-negative mutants, or small molecules induces apoptotic cell death in tumor cells but not in normal cells.^4^ Several chemical IAP antagonists, such as AT-406, LCL-161, GDC-0152, TL-32711, LBW242 and HGS-1029, which mimic the interactions of IAP proteins with secondary mitochondria-derived activator of caspase (SMAC) N-terminal peptide (an endogenous antagonist of IAP proteins), have been developed and are currently being evaluated in clinical settings.^5, 6, 7, 8^ The elucidation of the mechanism of antagonism and identification of biomarkers that indicate apoptotic cell death in tumors are key issues in the development of IAP antagonists. As such, the role of IAPs in regulating the apoptotic response and as molecular targets for achieving selective therapeutic effects in tumor cells has attracted great attention in an effort to identify peptide antagonists or small-molecule inhibitors.

Lung cancer is the leading cause of cancer-related death worldwide, with more than one million mortalities each year. Almost 85% of all lung cancer cases are diagnosed as non-small-cell lung cancers (NSCLC), which are further classified histologically as adenocarcinoma, squamous cell carcinoma or large cell carcinoma. Platinum-based chemotherapy represents the recommended standard first-line systemic treatment for advanced NSCLC, although the results of this approach are limited to a modest increase in survival rates. Epidermal growth factor receptor (EGFR) is often hyper-activated in many lung cancers due to the presence of a mutation in the kinase domain, causing the activation of multiple cell survival signals, especially Akt and mitogen-activated protein kinase (MAPK) pathways. This finding has led to the development of targeted therapeutics against the kinase, such as erlotinib and gefitinib, which becomes one of the most promising strategies for cancer treatment. The targeted therapeutics has often failed, however, due to the development of resistance through multiple mechanisms, indicating that additional adjuvants are necessary to achieve effective results.

In this study, we investigated the therapeutic potential of HM90822B, originally synthesized to inhibit IAP activity, on NSCLC cells and in a xenograft mouse model and analyzed the cellular effects of the drug to elucidate its mechanism of action. Our results showed that HM90822B inhibits cell growth resulting in cell cycle arrest and apoptosis by targeting XIAP and survivin in conjunction with the inhibition of EGFR-MAPK pathway, primarily AKT, p38 and c-jun phosphorylation. These results indicate that the IAP inhibitor HM90822B is a promising therapeutics for the treatment of NSCLC.

## Results

### NSCLC cells express high levels of the IAPs and EGFR

**Figure 1:**
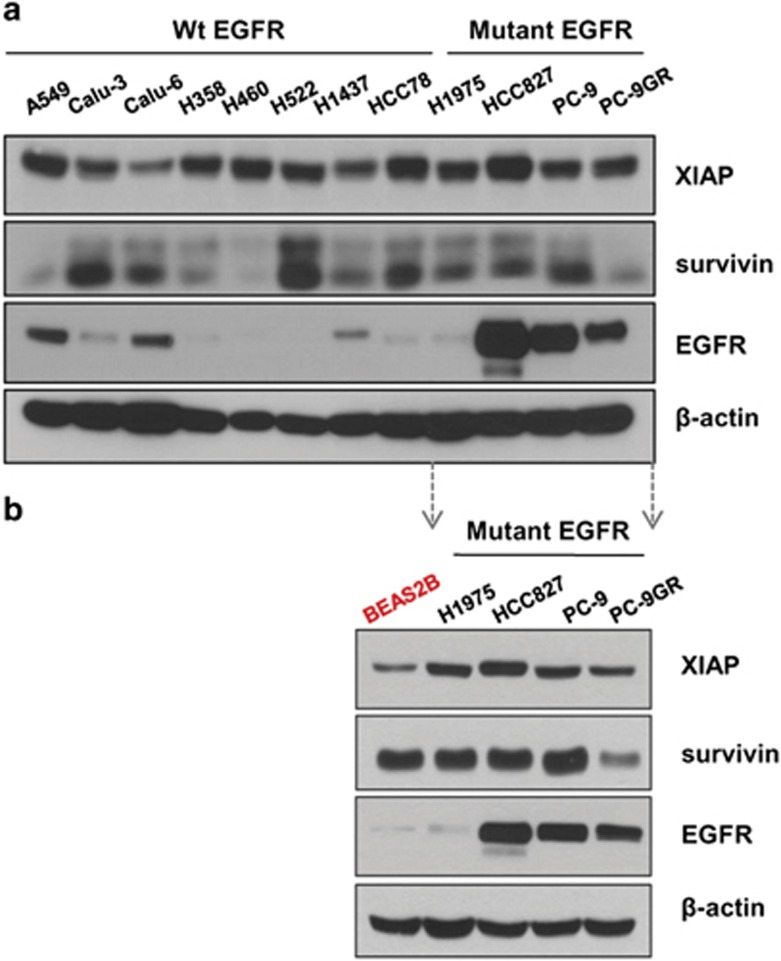
Endogenous protein expression in various NSCLC and Beas2B cell lines. Whole cell lysates (30 *μ*g) were analyzed by western blotting using antibodies against EGFR, XIAP, survivin and *β*-actin. (**a**) The protein expression was analyzed in NSCLC cells harboring wild type EGFR or mutant EGFR. (**b**) The protein expression in NSCLC cells harboring mutant EGFR was compared to that in immortalized normal lung cells, BEAS2B

The IAPs are highly expressed in a diverse array of tumors and are often associated with resistance to apoptosis and low sensitivity to chemotherapy drugs in some tumor types.^1, 2, 3^ Mutations and/or overexpression in EGFR that endow activated cell survival signaling have been regarded as a notorious cause of lung cancer and especially detected in almost half of NSCLC tumors. Among the many alterations that have been detected, L858R missense mutation or in-frame deletion at exon 19 are the most dominant, conferring hyper tyrosine kinase activity and stabilization of protein expression. To determine the expression levels of these oncogenic proteins in NSCLC cells, western blotting analyses were carried out in various NSCLC and immortalized lung cell lines (Figure 1). The IAP proteins, XIAP and survivin, were detected in almost all the cells tested; XIAP was highly expressed in all the 12 cell lines tested and survivin was also overexpressed in 8 out of the 12 NSCLC cell lines, confirming their tumorigenic effect in previous reports,^1,3^ although immortalized lung cell line, Beas2B, also expressed high level of survivin (Figure 1b). High levels of endogenous EGFR were detected in cells harboring exon 19 alterations, such as HCC827 (L858R mutation) and PC-9 (in-frame deletion) cells, whereas EGFR expression was barely detectable in cells harboring wild-type EGFR as well as H1975 cells containing L858R and T790M mutations.

### A novel synthetic small molecule targeting IAP family members, HM90822B, showed the inhibitory effect on human lung cancer cells

**Figure 2:**
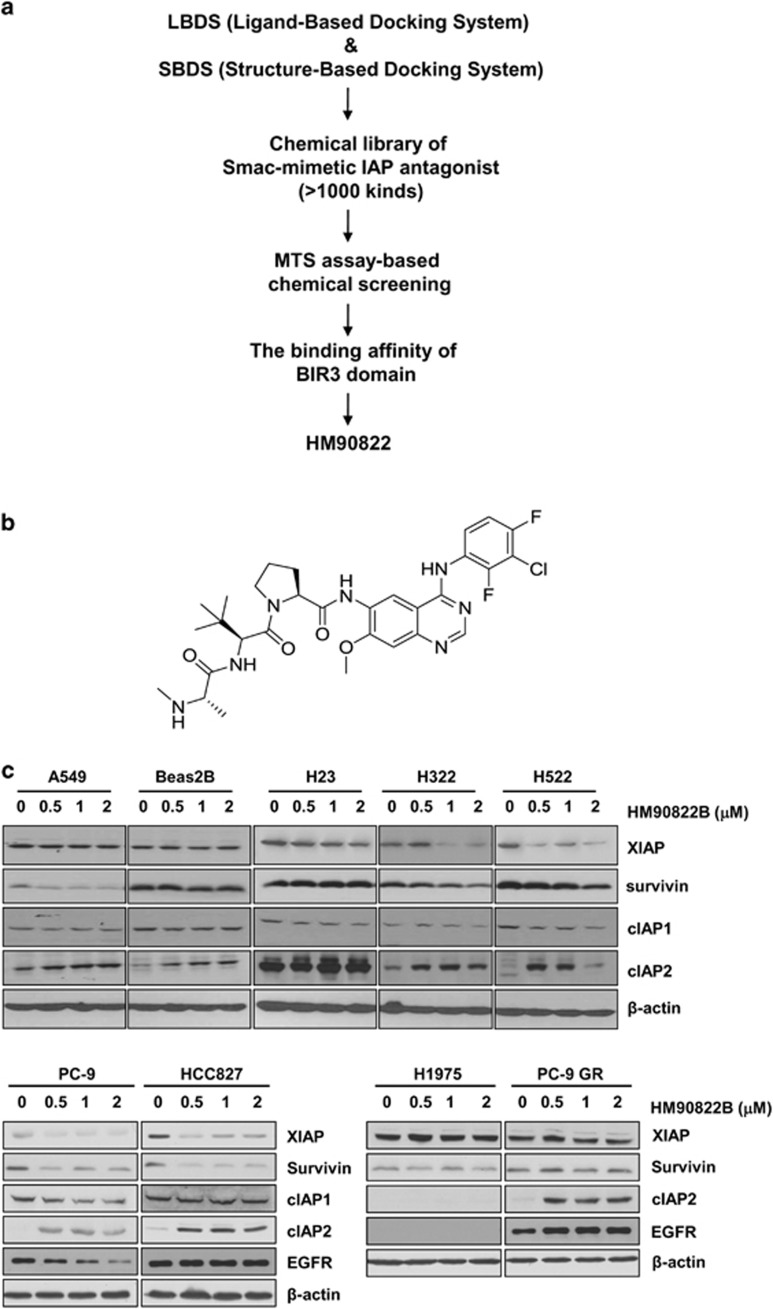
The selection procedure for the IAP inhibitor. (**a**) We first used LBDS (Ligand-Based Docking System) and SBDS (Structure-Based Docking System) to develop the small-molecule Smac-mimetic IAP antagonist, which was designed to bind to the BIR3 domain of IAP family members, as well as EGFR, and then screened compounds based on the biological effect by MTS (3-(4,5-dimethylthiazol-2-yl)-5-(3-carboxymethoxyphenyl)-2-(4-sulfophenyl)-2H-tetrazolium, inner salt) assay. (**b**) The structure of HM90822B. (**c**) The expression levels of IAPs were analyzed after treatment with HM90822B in NSCLC cells. A549, Beas2B, H23, H322 and H522 cells were incubated in the presence of the indicated concentrations of HM90822B for 3 days, and PC9, HCC827, H1975 or PC9GR cells were incubated in the presence of the indicated concentrations of HM90822B for 1 day. Whole cell lysates (30 *μ*g) were analyzed by western blotting with antibodies against XIAP, survivin, cIAP1, cIAP2, EGFR and *β*-actin

We first used LBDS (Ligand-Based Docking System) and SBDS (Structure-Based Docking System) to develop the small-molecule Smac-mimetic IAP antagonist, which was designed to bind to the BIR3 domain of IAP family members, as well as EGFR. To isolate more effective chemical in multiple human cancer cells that differently express the IAP family members, especially XIAP, cIAP1 and cIAP2, we performed MTS-based chemical screening using approximately 1000 kinds of chemicals. A group of chemicals showed the more inhibitory effect on human cancer cells, and we evaluated the binding affinity of BIR3 domain (Figure 2a). HM90822B, a new synthetic IAP antagonist, in a group of chemicals was found to bind the targeted domain with high affinity (*K*_*d*_=42.0 nM) (Figure 2b).

### HM90822B targets overexpressed IAPs in NSCLC cells

IAP proteins are overexpressed in many cancer cells, including NSCLC cells (Figure 1). To analyze the inhibitory effect of HM90822B on IAP proteins, we examined the protein expression levels of several IAP proteins after treatment with HM90822B in various NSCLC cells, including cells with EGFR tyrosine-kinase inhibitor (TKI)-resistant mutations, such as H1975 and PC-9GR (Figure 2c). HM90822B exhibited inhibitory effects on at least one subtype of IAP expression in each of the NSCLC cells tested; XIAP expression was decreased in H322, H522, PC-9 and HCC827 cells; survivin expression was decreased in A549, H322, H522, PC-9 and HCC827 cells; and cIAP1 expression was decreased in H322 and H522 cells. Interestingly, Beas2B cells, which originated from normal lung cells, showed no change in the expression level of XIAP, survivin or cIAP1 but an increase in the level of cIAP2. Unexpectedly, cIAP2 expression was also increased in many NSCLC cells tested, including H322, H522, PC-9, HCC827 and PC9GR cells. The expression level of XIAP or survivin, however, was unchanged in EGFR TKI-resistant H1975 and PC-9GR cells. The decreased level of EGFR was observed in PC-9 cells upon HM90822B treatment.

### HM90822B inhibits NSCLC cell growth

**Figure 3:**
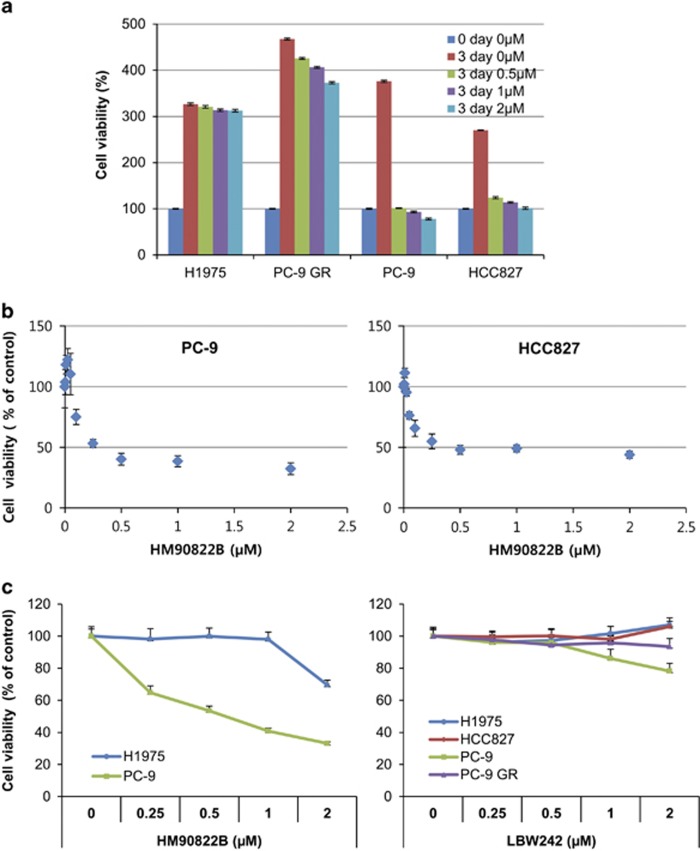
The effect of HM90822B on NSCLC cell growth. Cells were treated with increasing concentrations of HM90822B (up to 2 *μ*M). Cell proliferation was determined by MTT assay. The absorbance was measured on an ELISA plate reader (Molecular Devices) at a wavelength of 540 nm, and the inhibitory effects were normalized to untreated condition. (**a**) Growth graphs of NSCLC cells harboring mutant EGFR (H1975, PC-9 GR, HCC827 and PC-9). Cells were treated with increasing concentrations of HM90822B (up to 2 *μ*M) for 3 days. (**b**) Growth graphs of NSCLC cells harboring mutant EGFR sensitive to TKIs (HCC827 and PC-9). Cells were treated with increasing concentrations of HM90822B (up to 2 *μ*M) for 1 day. (**c**) Growth graphs of NSCLC cells. Cells were treated with increasing concentrations of HM90822B (up to 2 *μ*M) or LBW242 (up to 2 *μ*M) for 1 day

To explore whether decreased IAP expression affects NSCLC cell growth in response to HM90822B, cell proliferation was analyzed in NSCLC cells, as well as in MDA-MB-231 breast cancer cells. Cells were treated with various concentrations of HM90822B for 3 days, and their proliferation was determined by MTT assay. Pathologically different types of NSCLC cells were assessed, including H322 and H522, which contain wild-type EGFR, and H1975, PC-9 GR, HCC827 and PC-9, which contain mutant EGFR. H322 and H522 cells were relatively sensitive to HM90822B treatment, resulting in to 72 and 50% of growth rate at 2 *μ*M drug, respectively (Supplementary Figure S1A). Interestingly, the growth of PC-9 and HCC827 cells was dramatically affected upon 1 day treatment with HM90822B at a concentration of 0.5 *μ*M (Figure 3a). These cells responded even at lower concentrations, showing an IC_50_ of approximately 300 nM calculated on GraphPad Prism 6 (HCC827 cells, 321.7 nM; PC-9 cells, 314.9 nM) (Figure 3b). Conversely, H1975 and PC-9 cells were resistant to HM90822B (Figure 3a), indicating that EGFR expression level (Figure 1) may be an important factor for determining responsiveness to the drug. The effect of HM90822B on NSCLC cell growth was compared with the effect of known IAP inhibitor, Smac-mimetic LBW242, in the same concentration range up to 2 *μ*M for 1 day (Figure 3c). The results clearly showed that the novel IAP inhibitor would be a promising anti-tumor therapeutic.

### NSCLC cells treated with HM90822B undergo cell growth arrest and apoptosis

**Figure 4:**
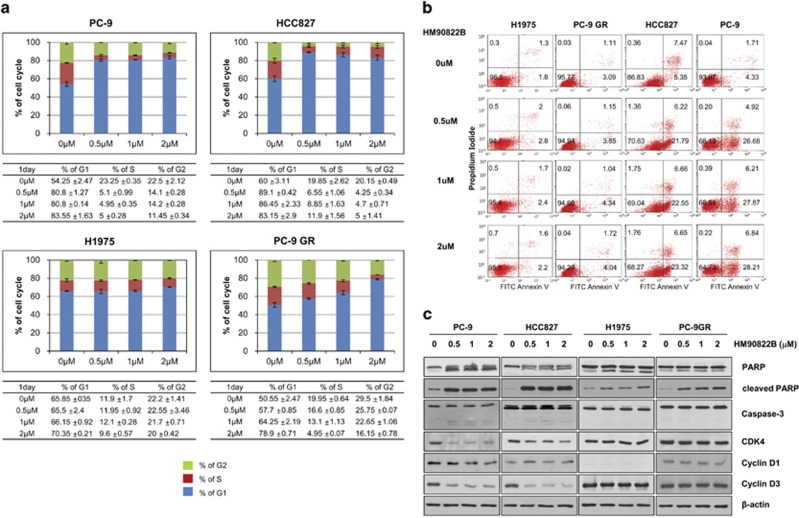
Cell cycle distribution and apoptosis in NSCLC cells after treatment with HM90822B. (**a**) PC9, HCC827, H1975 or PC9GR cells were incubated in the presence of the indicated concentrations of HM90822B for 1 day, fixed, stained with PI and analyzed for cell cycle distribution by flow cytometry. Each table for cell cycle distribution is attached under the graph for ease in interpretation. (**b**) Annexin V/PI staining assay was carried out to explore cell apoptosis induced by HM90822B. H1975, PC-9 GR, HCC827 and PC-9 cells were treated with 0, 0.5, 1 and 2 *μ*M HM90822B for 24 h, stained with Annexin V and PI and analyzed by flow cytometry. The data shown are representative of three independent experiments with similar findings. (**c**) Expression levels of apoptosis- or cell cycle-related proteins were decreased in NSCLC cells after treatment with HM90822B. PC9, HCC827, H1975 and PC-9GR cells were treated in the presence of the indicated concentrations of HM90822B for 1 day. Whole cell lysates were analyzed by western blotting using antibodies against PARP, cleaved-PARP, caspase3, CDK4, cyclin D1, cyclin D3 and *β*-actin

To identify the mechanism of HM90822B-mediated cell growth inhibition, we examined cell growth arrest and apoptosis after treatment with HM90822B. When cells were analyzed by FACS scan after treatment with various concentrations of HM90822B, HCC827 and PC-9 cells arrested at G1 were increased as much as 1.5-fold, although H1975 cells were not affected (Figure 4a). In all, 30% of HCC827 and PC-9 cells also underwent apoptosis, whereas <6% of H1975 and PC-9 GR cells did at 2 *μ*M of HM90822B treatment (Figure 4b). Proteins mediating cell cycle arrest and apoptosis were also examined by western blotting analysis after treatment with different concentrations (up to 2 *μ*M) of drug for 1 day (HCC827, PC-9, H1975 and PC-9GR in Figure 4c) or 3 days (H322 and H522 in Supplementary Figure S1B). Expression levels of CDK4 and cyclin D3 were decreased in drug-sensitive cells, especially in PC-9 and H522 cells, and not in drug-resistant cells. Cleavage of poly(ADP-ribose) polymerase (PARP) was clearly observed in drug-sensitive cells, although it was also observed in drug-resistant cells weakly. However, cleaved caspase-3 or the decreased level of pro-caspase-3 was detected in drug-sensitive cells, although the protein was not affected in H1975 or PC-9GR cells. Taken together, these results confirmed the cell cycle arrest and apoptosis results of the FACS analysis.

### HM90822B inhibits tumor growth in an NSCLC cell xenograft model

**Figure 5:**
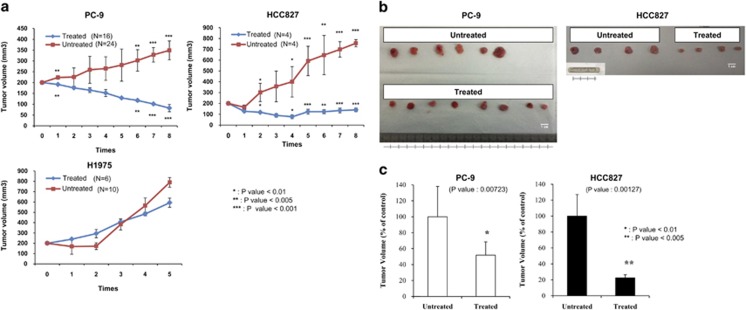
Effect of HM90822B on tumor growth in NSCLC cell xenografts. (**a**) PC-9, HCC827 and H1975 cells formed tumors of approximately 200 mm^3^ in size within 21 days of inoculation. Mice were then treated orally with PBS (denoted as untreated) or 100 mg/kg HM90822B (denoted as treated) for five times once every 2 days. Tumor volume was calculated using the formula for an ellipsoid sphere: *W*1 × *W*2 × *W*2/2=*x* mm^3^, where *W*1 represents the largest tumor diameter and *W*2 the smallest tumor diameter. Independent xenograft experiments were performed three times. *P*<0.01; *P*<0.005; *P*<0.001 compared with vehicle-treated tumors. (**b**) Representative tumors from PBS (untreated) or HM90822B (treated) mice were collected at the end points of the experiment. (**c**) Reduced tumor sizes in PC-9 and HCC827 cells were depicted graphically

We then examined the effect of HM90822B on NSCLC tumor growth *in vivo* using two representative drug-sensitive cell lines (HCC827, PC9) and one drug-resistant cell line (H1975). Cells were inoculated into athymic male nude mice (CD-1 *nu*/*nu* nude mice), and when tumors grew into approximately 200 mm^3^ in size (21 days), the mice were treated with phosphate-buffered saline (PBS) or 100 mg/kg of HM90822B orally for five times once every 2 days. Tumors formed from HCC827 and PC9 NSCLC cells, which harbor activated EGFR signaling resulting from mutations in exon 21 and exon 19, respectively, were sensitive to HM90822B treatment and shrank by approximately eight-fold compared with tumors in mice treated with PBS. However, tumors from H1975 cells, which contain the additional drug resistance mutation T790M, showed no difference in tumor size upon HM90822B treatment (Figure 5a). Representative tumors taken from mice treated with HM90822B showed obvious shrinkage compared with untreated mice (Figure 5b) and is depicted graphically (Figure 5c). These results demonstrated that HM90822B has an anti-tumor effect *in vivo* in certain types of lung tumors.

### MAPK signaling in NSCLC cells is affected by treatment with HM90822B

**Figure 6:**
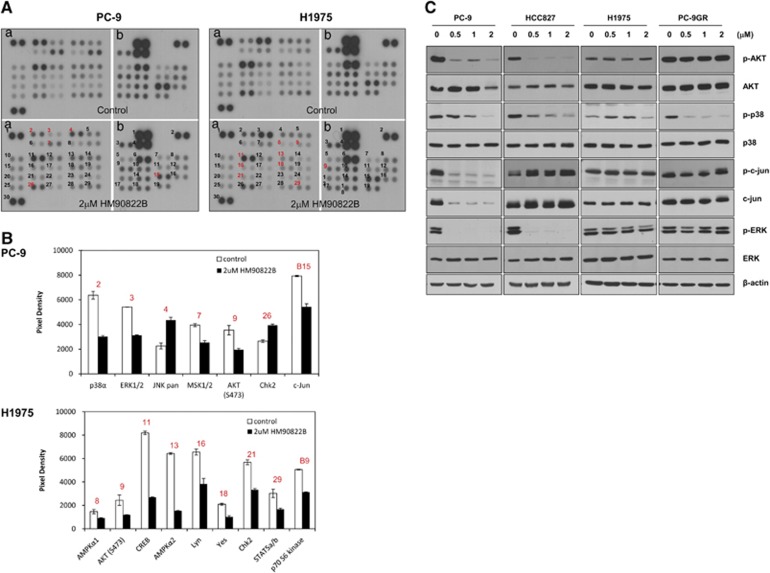
Phosphorylation array in PC-9 and H1975 cells after treatment with HM90822B. (**A**) Cells were treated with PBS (control) or HM90822B (2 *μ*M) for 24 h, and the cell lysates were analyzed by phosphorylation array. (**B**) The degree of phosphorylation of each protein was determined by comparing the phosphorylation intensity in treated cells with that in untreated cells. (**C**) Phospho-proteins affected by HM90822B treatment, phosphor-Akt, phosphor-p38, phosphor-c-Jun and phosphor-Erk, were confirmed in NSCLC cells by western blotting analysis

It is interesting that a novel drug HM90822B showed cell growth-inhibitory effect on NSCLC cells, especially on IAP and EGFR-overexpressing cells, because both molecules are regarded as oncogenic and tumorigenic in different types of cancers. To understand the working mechanism of HM90822B on cell proliferation, we assessed changes in signaling proteins using a Human Phosph-Kinase array panel containing 58 kinases (listed in Supplementary Table S1). The phosphorylation changes in H1975 (HM90822B-resistant) and PC-9 (HM90822B-sensitive) cells were assayed before and after treatment with 2 *μ*M of the drug for 24 h. Although Akt phosphorylation (S473) was decreased in both H1975 and PC-9 cells, phosphorylations of proteins in MAPK pathway, such as ERK, p38 and c-Jun, were affected only in PC-9 cells (Figures 6A and B), suggesting that HM90822B inhibits cell growth by attenuating MAPK signaling. These alterations were confirmed in western blotting analysis to visualize the effects of HM90822B on the signal pathways in NSCLC cells (Figure 6C). The kinases, AKT, p38, ERK and c-Jun, were examined. Phosphorylations of AKT, p38, ERK and c-Jun were decreased upon treatment of HM90822B in PC-9 cells although the protein expression of c-Jun was also decreased. HCC827 cells showed the decreased levels of phosphor-AKT, -p38 and -ERK. However, H1975 cells showed no difference in the expression of proteins tested, and PC-9GR cells showed the decreased level of phosphor-p38 only (Figure 6C). Phosphorylation of Erk was also reduced in H322 and H522 cells (Supplementary Figure S1B). Together, these results implied that HM90822B could control the cell survival signaling proteins to elicit NSCLC cell growth inhibition.

### Downregulations of EGFR and XIAP expression inhibit NSCLC cell growth synergistically with HM90822B treatment

**Figure 7:**
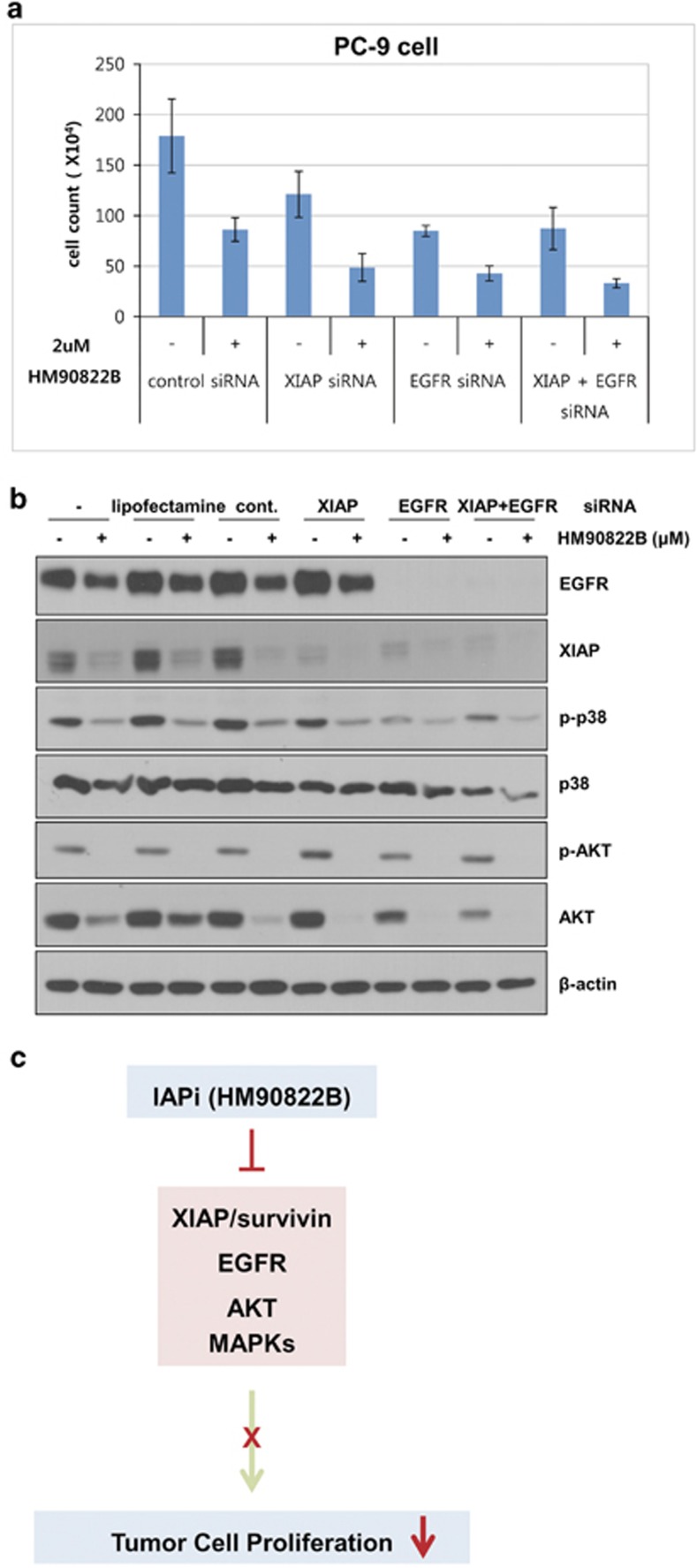
Knockdown of XIAP and EGFR expression in PC-9 cells. Cells were transfected with 50 nM XIAP siRNA, EGFR siRNA or negative control siRNA for 24 h and then incubated with 2 *μ*M HM90822B for 24 h. (**a**) Cell growth was determined by counting trypan-blue unstained cells. (**b**) The expression levels of EGFR, XIAP, phosphor-p38, p38, phosphor-AKT, AKT and *β*-actin were determined by western blotting analysis. (**c**) Working model was depicted for cell growth-inhibitory effect of HM90822B in NSCLC cells

To further determine the role of XIAP and EGFR on cell growth-inhibitory effect of HM90822B in NSCLC cells, we downmodulated these gene expression levels with gene-specific siRNAs in PC-9 NSCLC cells, in combination with HM90822B. Cells were transfected with 50 nM XIAP siRNA, 10 nM EGFR siRNA or 50 nM negative control siRNA for 24 h and incubated with 2 *μ*M HM90822B for 24 h, and then the cell numbers were counted (Figure 7a). Reduced expression of EGFR and XIAP was determined by western blotting analysis using anti-EGFR and anti-XIAP antibodies (Figure 7b). PC-9 cell growth was reduced to 45% with control siRNA transfection and HM90822B treatment. When XIAP or EGFR siRNA was treated, cell growth was further decreased, demonstrating that both proteins had critical roles in NSCLC cell growth. There was no additive effect of two molecules on cell growth (Figure 7b). Moreover, when EGFR was knocked down, the expression of XIAP was dramatically affected (Figure 7b), implying that two molecules are on the same signaling pathway. Importantly, addition of HM90822B to XIAP or EGFR siRNA-transfected PC-9 cells further induced retardation of cell growth, indicating additive effect of downmodulation of each protein with HM90822B. This effect was exaggerated by treatment with all three components (Figure 7a). The expression levels of proteins involved in cell survival signaling were assessed by western blotting analysis, showing that phosphorylations of p38 and Akt were dramatically affected by HM90822B treatment but barely and slightly reduced through siRNA treatment of XIAP and EGFR, respectively (Figure 7b). This result implied that the effect of HM90822B on cell growth was mediated not only through these two oncogenic molecules but also via other mechanism, such as inhibition of Akt/MAPK signaling pathways, in accordance with the results in the phosphor-kinase array and western blotting analysis shown in Figure 6. Together, these results suggest that the effect of HM90822B on cell growth might occur through diverse mechanisms, leading to hypothetical working pathway of this novel drug as in Figure 7c that may be proved with ongoing further studies.

## Discussion

Although therapeutics targeted to activated EGFR have contributed significantly to the management of NSCLC, long-term treatment with these drugs often leads to the development of drug resistance and tumor relapse.^9^ The IAPs are key regulators of apoptosis and are often overexpressed in tumors that are drug-resistant. These proteins are also associated with disease progression and poor prognosis, making them promising targets for cancer therapeutics designed to treat many types of solid cancers, including lung cancer.^10,11^ In this study, we found that a novel IAP inhibitor, HM90822B, originally designed to target IAP family and EGFR and screened as an IAP inhibitor, greatly sensitized several NSCLC cells to apoptosis and cell growth arrest. As reported in other types of tumors, IAP proteins, especially XIAP and survivin, were highly expressed in many of the NSCLC cell lines tested in this study, emphasizing that some of NSCLC cell lines overexpress both IAPs and EGFR, suggesting that both oncogenic molecules are suitable targets for the treatment of lung cancer. We found that HM90822B significantly inhibited the expression of XIAP and survivin, reduced cell growth in NSCLC cells, especially cells harboring mutated EGFR, and resulted in tumor regression in xenograft mice originated with these cells, such as HCC827 and PC-9 cells. However, HM90822B was unable to halt the growth of tumors formed from cells with an additional T790M (EGFR TKI-resistant) mutation and less expression of EGFR. HM90822B induces cell growth arrest by inhibiting CDK4 and cyclin D3 in NSCLC cells. Mechanistically, the anti-proliferation effect of the IAP inhibitor was dependent on the inhibition of MAPK pathways, especially p38 and c-Jun, as well as on decreased phosphorylation of Akt and Erk. Downregulation of XIAP or EGFR expression decreased cell growth in PC-9 cells, indicating that these molecules have critical roles in cell proliferation. Interestingly, co-treatment of siRNA and drug augmented the inhibitory effect on NSCLC cells, suggesting that diverse mechanisms are involved. HM90822B, nevertheless, had little effect on the expression of cIAP2, implying that inhibition of one or more IAPs together with EGFR is critical in the growth of NSCLC cells. Our results demonstrate that targeting both oncogenes with one agent represents promising approach to cancer therapeutics.

Phosphorylation of XIAP by activated Akt is known to accelerate the anti-apoptotic activity of this IAP. Akt is involved in cell survival and proliferation in various tumor types and can be phosphorylated and activated through several signaling pathways, including MAPK and EGFR signaling. NSCLC tumorigenesis mediated by increased EGFR activity due to its overexpression or mutation is associated with poor prognosis and advanced tumorigenesis. Therefore enormous efforts have been focused on targeting EGFR signaling.

Many studies have explored the mechanism by which members of the IAP family regulate apoptotic processes. XIAP acts as a direct caspase inhibitor, and cIAP1 and cIAP2 proteins protect cells from apoptosis by primarily acting as a E3 ubiquitin ligase through C-terminal RING finger domains.^12^ The role of cIAPs in preventing apoptosis is reported to be via mediating proteosomal degradation of caspases 3 and 7.^13^ Binding and phosphorylation of XIAP at serine-87 residue by AKT is known to protect XIAP from ubiquitination and degradation in response to cisplatin. Moreover, AKT also inhibits auto-ubiquitination.^14^ The inhibitor HM90822 may provide advantages for the reduction of tumor growth by inhibiting AKT followed by accelerating phosphorylation and auto-ubiquitination of XIAP, which would be mechanistically uncovered in further ongoing studies. The fact that many cancer cells express elevated levels of IAPs and escape apoptosis indicates that IAPs are good targets for the development of cancer therapeutics. The activity of Smac, an IAP-inhibiting mitochondrial protein, was confirmed to improve the survival of xenografted mice injected with pancreatic cancer cells.^15, 16, 17^ Several other small molecules have also been developed for use as anticancer therapeutics and act by antagonizing the activities of XIAP,^18^ survivin^19,20^ and cIAP.^21, 22, 23^ Some of these inhibitors are currently in Phase I/II clinical trials, including the survivin antisense compound LY-2181308 (Eli Lilly/Isis Pharmaceuticals), the XIAP antisense compound AEG35156 (Aegera Therapeutics), the small-molecule survivin antagonist YM-155 (Astellas Pharma) and the IAP inhibitor HGS1029 (Human Genome Sciences).

To improve the anti-tumor effects of these drugs, combination treatment of various types of tumors has been tested. EGFR inhibitors, in combination with the conventional chemotherapeutic drug cisplatin, prevented the development of resistance to cisplatin-mediated apoptosis via the phosphatidylinositol 3-kinase/Akt pathway and modulated the activity of apoptosis-related proteins such as IAPs and members of the Bcl-2 family. This illustrates an example of the therapeutic advantage of co-treatment with the benefit of reduced toxicity of conventional drugs.^24^ Previously, RNAi-mediated inhibition of IAPs in combination with ErbB antagonists, such as the EGFR inhibitor gefitinib, profoundly enhanced apoptosis in breast cancer cells.^25^ However, although some instances of single small molecules affecting multiple targets have been reported, none have been shown to inhibit EGFR and IAPs.^26^

The present study showed, for the first time, the concept of simultaneously inhibiting dual targets in NSCLC. We showed that the antagonist HM90822B efficiently downregulates XIAP and survivin in human EGFR-overexpressing NSCLC cells and demonstrated that this downregulation contributes to enhance cell cycle arrest and apoptosis, even to regress tumor growth *in vivo*. The current results complement previous findings that combined inhibition of EGFR signaling and the IAP-mediated anti-apoptotic pathway could fulfill a critical role in the treatment of human NSCLC cells, and ongoing further studies may provide better understanding for the working mechanism of this novel drug.

## Materials and Methods

### Human cancer cell lines, chemicals and reagents

The human breast cancer cell line MDA-MB-231 and the NSCLC cell lines A549, Beas2B, H23, H322, H522, H1975, PC-9, HCC827 and PC-9GR were obtained from the American Type Culture Collection (Manassas, VA, USA). Cells were maintained in RPMI 1640 or DMEM supplemented with 10% fetal bovine serum, 2 mM L-glutamine, 100 IU/ml penicillin and 100 *μ*g/ml streptomycin (Invitrogen, Carlsbad, CA, USA) in a humidified environment containing 5% CO_2_. Synthetic IAP antagonists HM90822B and LBW242 were supplied by Hanmi Pharm. Co., Ltd. (Seoul, Korea).

### Cell proliferation analysis (MTT assay)

Cancer cells were treated with different concentrations of HM90822B or LBW242 in DMEM supplemented with 10% FBS. Cell proliferation was measured with the (3-(4, 5-dimethylthiazol-2-yl)-2, 5-diphenyltetrazolium bromide (MTT) assay as previously described.^27^

### Cell cycle and apoptosis assays

For cell cycle and apoptosis assays, both adherent and non-adherent cells were harvested, pooled and fixed with 90% methanol. For cell cycle analysis, cells were stained with a solution containing 50 *μ*g/ml propidium iodide (PI) and 30 *μ*g/ml RNase A in 1 × PBS. The percentage of cells in specific cell cycle phases (G1, S and G2/M) was determined using a flow cytometer equipped with a 488-nm argon laser (FACS Calibur, BD Biosciences, San Jose, CA, USA). Approximately, 1 × 10^4^ cells were evaluated for each sample. Apoptosis was analyzed by double staining with Annexin V-FITC and PI using an Annexin V-FITC apoptosis detection kit (BD Biosciences) following the manufacturer's recommended protocol.

### Western blotting analysis

Total protein was isolated and subjected to western blotting analysis as described previously. The following antibodies were used for the western blottings: rabbit polyclonal antibodies detecting XIAP, cIAP1, cIAP2, survivin, cyclin D1, CDK4, Akt, p44/p42 MAPK (Erk) and c-jun (Santa Cruz Biotechnology, Santa Cruz, CA, USA); EGFR, cyclin D3, phospho-serine473 Akt, phospho-p44/p42 MAPK (Erk), cleaved PARP (p85), caspase-3, phospho-p38, p38 and phospho-c-jun (Cell Signaling Technology Inc., Danvers, MA, USA); and *β*-actin (Sigma Chemical Co., St. Louis, MO, USA). After incubation with the corresponding secondary antibodies for 1 h, signals were detected using an Enhanced ChemiLuminescence kit (Thermo Fisher Scientific, Inc., Rockford, IL, USA) followed by autoradiography.

### Mouse xenograft cancer model

Athymic male nude mice (CD-1 *nu*/*nu* from the Jungang Inc., Seoul, Republic of Korea) were used for *in vivo* tumor growth studies. All of the *in vivo* studies were carried out following the approved institutional experimental animal care and use protocols. For the NSCLC tumor model, HCC827, PC9, or H1975 cells were suspended at 5 × 10^6^ cells per 200 *μ*l in PBS and injected into the legs of 4-week-old athymic nude mice. When the tumors were approximately 200 mm^3^ in size (21 days), the animals were randomly allocated into four groups. Following randomization, vehicle or 0.2 ml of HM90822B (100 mg/kg, equivalent to a human dose of 8 mg/kg by normalization to the surface area) were delivered orally for five times once every 2 days for the duration of the experiment. Tumor size was measured with digital calipers 3 times weekly. Tumor measurements were converted to tumor volume using the following formula: *W*1 × *W*2 × *W*2/2=*x* mm^3^ (where *W*1 represents the largest tumor diameter and *W*2 the smallest tumor diameter, respectively). Mice were killed when the tumors exceeded 15 mm^3^ in the control group, and tumors were excised and assessed histologically for verification of tumor growth. Statistical significance was determined by the Student's *t*-test.

### Phosphorylation array

Human Phospho-Kinase Array Kits (R&D Systems, Minneapolis, MN, USA) were used to measure protein phosphorylation according to the manufacturer's protocol. Briefly, 1 × 10^6^ cells were plated in culture plates and incubated overnight. Following a 24-h exposure to 2 *μ*M HM90822B, the cells were rinsed once with PBS and solubilized with lysis buffer. A total of 600 *μ*g protein was applied to the array. Membranes were then scanned, and the density was measured.

### SiRNA transfection

Cells in six-well plates were transfected with siRNAs and Lipofectamine 2000 (Invitrogen, Grand Island, NY, USA) in Opti-MEM (Life Technologies Inc., Grand Island, NY, USA) without serum, according to the manufacturer's specifications. After incubation for the indicated periods, cell proliferation was measured by counting trypan-blue unstained cells, and protein expression was analyzed by western blotting. The siRNAs used were generated from Bioneer Corp. (Daejeon, Republic of Korea) according to previous reports, and the sequences are as follows: XIAP 5′-GUAAAAUGCAAGUGGCAAAUU-3′^28^ and EGFR 5′-UUUAAAUUCACCAAUACCUAUUCCG-3′.^29^

## Abbreviations

IAP: inhibitor of apoptosis
EGFR: epidermal growth factor receptor
NSCLC: non-small-cell lung cancer
BIR: baculoviral IAP repeat
RING: really interesting new gene
XIAP: X-linked IAP
cIAP: cellular IAP
MAPK: mitogen-activated protein kinase
siRNA: short interfering RNA
TKI: tyrosine-kinase inhibitor
Smac: secondary mitochondria-derived activator of caspase
