# Supplementary material for: A novel antagonist to the inhibitors of apoptosis (IAPs) potentiates cell death in EGFR-overexpressing non-small-cell lung cancer cells
Source: Cell Death Dis. 2014 Oct 16;5(10):e1477–. doi: 10.1038/cddis.2014.447 (PMC4649530; doi:10.1038/cddis.2014.447)
Supplement: Supplementary Table S1 [file cddis2014447x1.doc]

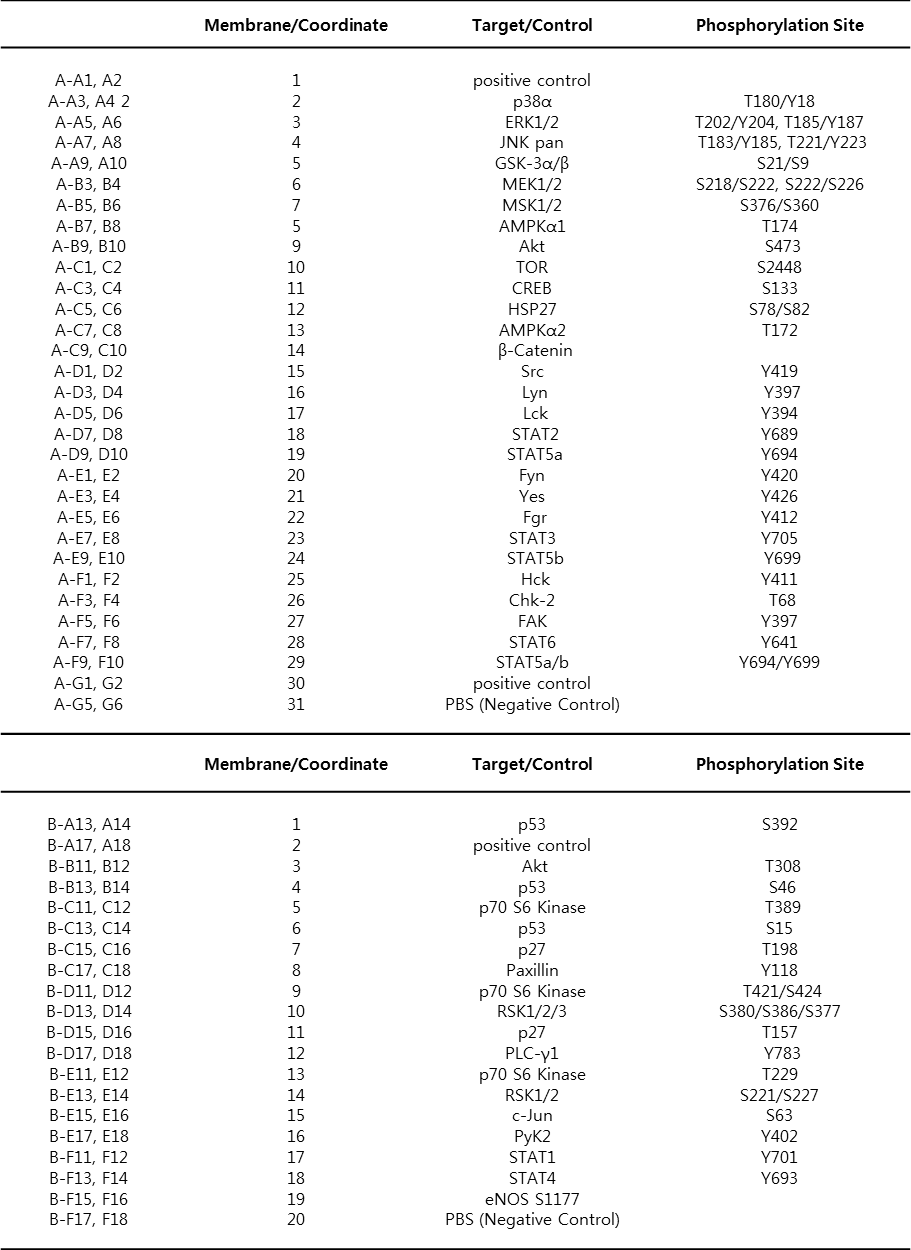
Supplementary Table 1. Proteins and their phosphorylation sites on Human Phospho-Kinase Array Kits (R&D Systems)
